# Macrophage Phenotypes and Gene Expression Patterns Are Unique in Naturally Occurring Metabolically Healthy Obesity

**DOI:** 10.3390/ijms232012680

**Published:** 2022-10-21

**Authors:** Alistaire D. Ruggiero, Ravichandra Vemuri, Masha Block, Darla DeStephanis, Matthew Davis, Jeff Chou, Abigail Williams, Ashlynn Brock, Swapan Kumar Das, Kylie Kavanagh

**Affiliations:** 1 Department of Pathology, Wake Forest University School of Medicine, Winston-Salem, NC 27157, USA; 2 Department of Internal Medicine, Wake Forest University School of Medicine, Winston-Salem, NC 27157, USA; 3 Department of Biostatistics and Data Science, Wake Forest University School of Medicine, Winston-Salem, NC 27157, USA; 4 Department of Endocrinology and Metabolism, Wake Forest University School of Medicine, Winston-Salem, NC 27157, USA; 5 College of Health and Medicine, University of Tasmania, Hobart 7000, Australia

**Keywords:** obesity, metabolic syndrome, adipose tissue, macrophages

## Abstract

Obesity impacts 650 million individuals globally, often co-occurring with metabolic syndrome. Though many obese individuals experience metabolic abnormalities (metabolically unhealthy obese [MUO]), ~30% do not (metabolically healthy obese [MHO]). Conversely, >10% of lean individuals are metabolically unhealthy (MUL). To evaluate the physiologic drivers of these phenotypes, a 44-animal African green monkey cohort was selected using metabolic syndrome risk criteria to represent these four clinically defined health groups. Body composition imaging and subcutaneous adipose tissue (SQ AT) biopsies were collected. Differences in adipocyte size, macrophage subtype distribution, gene expression, vascularity and fibrosis were analyzed using digital immunohistopathology, unbiased RNA-seq, endothelial CD31, and Masson’s trichrome staining, respectively. MHO AT demonstrated significant increases in M2 macrophages (*p* = 0.02) and upregulation of fatty acid oxidation-related terms and transcripts, including *FABP7* (*p* = 0.01). MUO AT demonstrated downregulation of these factors and co-occurring upregulation of immune responses. These changes occurred without differences in AT distributions, adipocyte size, AT endothelial cells, collagen I deposition, or circulating cytokine levels. Without unhealthy diet consumption, healthy obesity is defined by an increased SQ AT M2/M1 macrophage ratio and lipid handling gene expression. We highlight M2 macrophages and fatty acid oxidation as targets for improving metabolic health with obesity.

## 1. Introduction

More than 1.9 billion adults worldwide are overweight (BMI: 25–29.9 kg/m^2^), and over 650 million are obese (BMI ≥ 30 kg/m^2^) [1]. Obesity often coincides with metabolic syndrome (MetS), which is defined as the presence of two or more of the following: increased waist circumference, hypertriglyceridemia, low high-density lipoprotein (HDL) cholesterol, elevated fasting glucose, and/or hypertension [2]. However, roughly 30% of obese people remain free of MetS [3], while over 10% of lean individuals demonstrate metabolic abnormalities [2]. The mechanisms that sustain the metabolically healthy obese (MHO) or propagate the metabolically unhealthy lean (MUL) phenotypes are undetermined.

The MHO and MUL phenotypes are evidence that complex interactions exist between adipose tissue (AT) and metabolic regulation. Macrophages constitute 5% of white AT cell content in healthy lean states and up to 50% of cell content in obese states [4]. As the predominant leukocyte in AT, macrophages respond to stimuli in their microenvironments and range from highly pro-inflammatory to highly anti-inflammatory [5]. While heterogeneous, AT macrophages are often defined by the extremes of their inflammatory states [5]. Recruited M1, or classically activated, macrophages are prompted by Toll-like receptor (TLR) ligands [6,7], and demonstrate high antigen presentation and expression of pro-inflammatory cytokines [7]. Embryo-derived M2 macrophages, or alternatively activated macrophages, are induced by anti-inflammatory stimuli [7]. Intermediate macrophages express M1 and M2 markers, and both associate with insulin resistance and demonstrate increases in mitochondria and upregulation of fatty acid metabolism enzymes [8]. Excess M1 macrophage infiltration results in increased tissue inflammation, while an overabundance of M2 macrophages can cause aberrant fibrosis [9]. AT macrophages display variable metabolic characteristics in situ based on their phenotypes. Adipose M2 macrophages may interact beneficially with adipocytes due their ability to release catecholamines and induce local lipolysis, and accept intercellular transfer of mitochondria from neighboring adipocytes [10,11,12]. They are also better suited for fueling oxidative phosphorylation using locally produced fatty acids [10,11,12]. To date, the spectrum of macrophages present in AT has not been determined in all four metabolic health groupings. Further, macrophage types are most commonly studied as part of the high-fat and high-sugar diet-driven MUO phenotype resulting in M1 macrophages and inflammation dominating the literature [13].

The purpose of this work was to identify underlying AT differences in distributions, macrophage profiles and gene expression in these four metabolic health groups. Here, we evaluated SQ AT differences between metabolic phenotypes (metabolically healthy lean [MHL] vs. MHO, MUL, and metabolically unhealthy obese [MUO]) that occur spontaneously without unhealthy diet exposure or changes in the environment. To do this, we used a unique nonhuman primate (NHP) model, the African green monkey (*Chlorocebus aethiops sabaeus*). We employed standard immunohistochemical approaches to evaluate macrophage subtypes and unbiased RNA-seq to evaluate subcutaneous AT transcriptional differences between metabolic health phenotypes. In doing so, we determined that MHO SQ AT is uniquely different in its macrophage cell polarization and metabolic gene expression signatures.

## 2. Results

### 2.1. Abdominal Adipose Tissue Distribution by Depot, Adipocyte Size, and Circulating Insulin Do Not Explain Poor Metabolic Health

**Figure 1:**
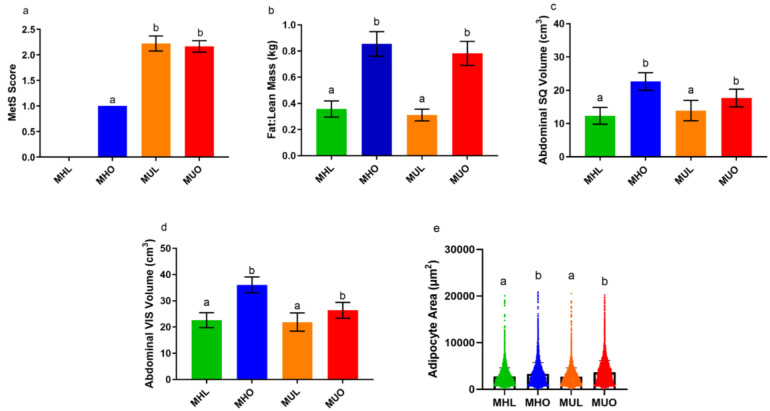
Nonhuman primate cohort body composition and adipocyte sizes. All values are presented as means ± SEM. Factorial ANOVA and ANCOVA with Tukey post hoc analyses were used to determine differences by obesity status and health status. Unlike letters denote statistically significant differences (*p* < 0.05) by health status or obesity status, specifically between groups labeled “a” versus “b”. Groups labeled with the same letter were not statistically different. (**a**) Metabolic syndrome scores for each health group (*p* = 0.00016; MHL, *n* = 12; MHO, *n* = 10; MUL, *n* = 9; MUO, *n* = 13). Differences in (**b**) fat to lean mass ratio (*p* ≤ 0.0001), (**c**) abdominal subcutaneous (SQ) adipose volume (*p* = 0.00023), and (**d**) abdominal visceral (VIS) adipose volume (*p* = 0.0095) between health groups. (**e**) Histological quantification of adipocyte sizes (*p* = 0.00056). The violin plot provided illustrates the distribution of adipocyte sizes in each health group. The cross bars indicate the mean adipocyte size value for each group.

By design, obese animals (MHO and MUO) were selected to have increased waist circumferences (Table 1). On average, obese animals weighed 1.6 kilograms more, and their average body fat was 19 percent higher than lean animals (Table 1). One point was allotted for each MetS risk criteria (cut-off values defined in Supplementary Table S1). In line with human clinical categorizations, MHL animals had a MetS score of zero, MHO animals had a MetS score of one for their obesity status, and MUL and MUO animals had MetS scores of two or higher (Figure 1a) [14]. By design, the MHO and MUO animals demonstrated increased waist circumferences compared to the MHL and MUL groups. MUL and MUO animals demonstrated increased fasting glucose values, percent glycosylated hemoglobin), systolic and diastolic blood pressure, and triglycerides. Computed tomography (CT) data concluded that obese animals exhibited increased percent fat mass and decreased percent lean mass. However, CT image analysis revealed no differences in fat-to-lean mass ratio, or abdominal SQ or visceral (VIS) adipose volume by health status (Figure 1b–d). Obese animals demonstrated lower VIS:SQ ratios than lean animals, though values between MHO and MUO animals were not different (Table 1). Lean animals demonstrated significantly less SQ and VIS adipose. The substantially lower amounts of SQ adipose drove the high VIS:SQ ratios in lean animals. Obese animals also had decreased percentage of lean mass body weight (Table 1). While food intake and caloric expenditure were not measured, percent lean mass, a measure of primarily muscle, was comparable between the MHO and MUO groups. Additionally, no differences in liver adipose, estimated as liver attenuation values, were identified (Table 1). MHL and MHO nonhuman primate (NHP) circulating insulin concentrations were comparable (Table 1) in contrast to MUO animals, which demonstrated significantly increased circulating insulin concentrations. MUL animals demonstrated significantly increased systolic and diastolic blood pressures. Histological evaluation of SQ adipose illustrated that differences in adipocyte cell size was driven by obesity status alone, where obese animals displayed larger SQ adipocytes regardless of their health status (Figure 1e; Supplementary Figures S1 and S2). Adipocyte size did not associate with metabolic syndrome-related clinical parameters.

### 2.2. Metabolically Healthy Obese Subcutaneous Adipose Demonstrates Increased Anti-Inflammatory M2 Macrophage Accumulation

**Figure 2:**
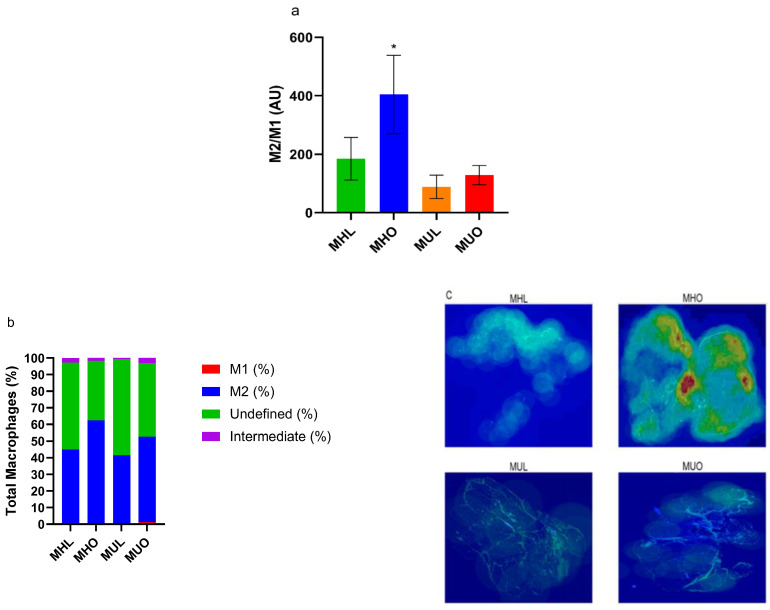
Metabolically healthy obese subcutaneous adipose demonstrates increases in anti-inflammatory M2 macrophages. (**a**) The ratio of M2/M1 macrophages between metabolic health groups (overall analysis of variance (ANOVA) *p* = 0.02; health effect *p* = 0.007). M2/M1 ratios are presented as means ± SEM (metabolically healthy lean (MHL), *n* = 12; metabolically healthy obese (MHO), *n* = 10; metabolically unhealthy lean (MUL), *n* = 9; metabolically unhealthy obese (MUO), *n* = 13). * Indicates Tukey post hoc significance of *p* < 0.05 compared to all other groups. (**b**) Stacked bars of the macrophage subtype percentages found in the SQ AT of each group. (**c**) Representative M2 macrophage AT heat maps, where increasing green to red coloration indicates higher densities of M2 macrophages.

We assessed tissue inflammatory profiles by characterizing SQ AT macrophage subtypes. Supplementary Figure S2 illustrates the staining combinations used to identify undefined, M1, intermediate, and M2 macrophages. SQ total AT macrophage density was comparable between all groups (Supplementary Table S2); however, MHO animals demonstrated significantly higher M2/M1 macrophage ratios (*p* = 0.02; Figure 2a–c). Differences in specific macrophage subtypes did not reach statistical significance (Supplementary Table S2), highlighting the importance of the M2/M1 macrophage ratio in local and systemic inflammation. Anti-inflammatory M2 macrophages were negatively associated with circulating levels of monocyte chemoattractant protein (MCP)-1 (r = −0.34, *p* = 0.03; Supplementary Figure S3). Though this association is moderate, it indicates that the healthy SQ AT milieu likely initiates less macrophage recruitment for harmful inflammation resolution. MUO animals demonstrated the highest percentage of both pro-inflammatory M1 and intermediate macrophages (Figure 2b; Supplementary Table S1). Notably, a large percentage of SQ adipose macrophages in each group were undefined or not yet polarized (Figure 2b).

### 2.3. Metabolically Healthy Obese Subcutaneous Adipose Demonstrates Upregulation of Lipid Handling-Related Transcripts without Immune Activation

**Figure 3:**
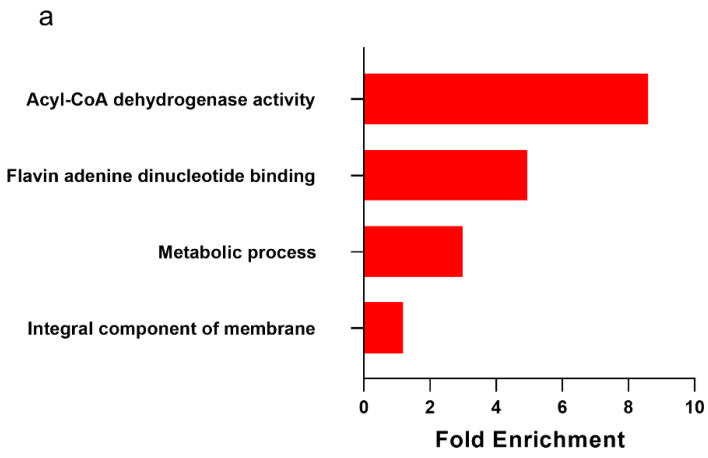

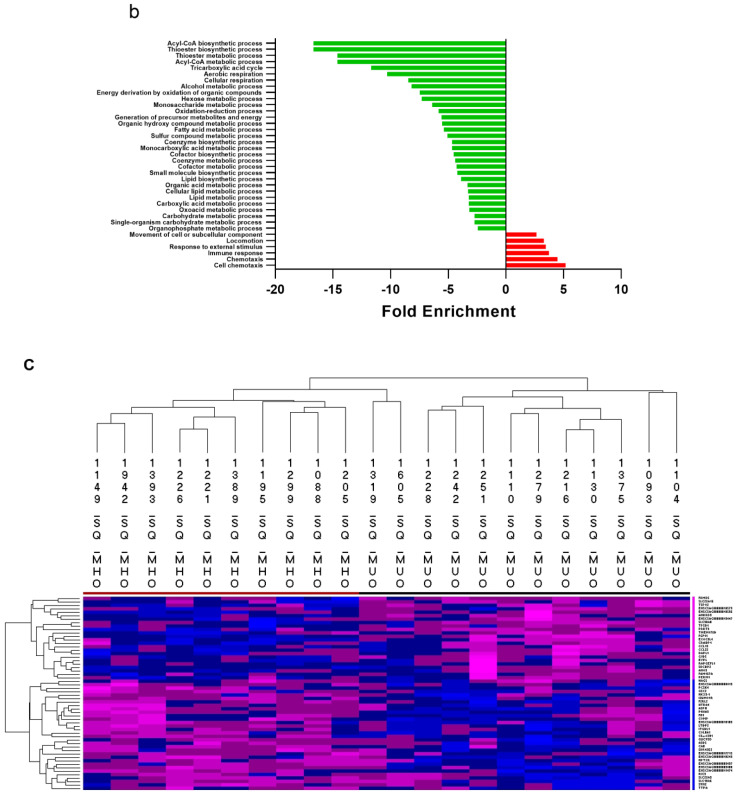
Metabolically healthy obese subcutaneous adipose illustrates increases in fatty acid oxidation-related gene ontology terms and downregulation of pro-inflammatory transcripts. Red coloration indicates pathway upregulation while green coloration indicates pathway downregulation. Pathways shown all survived false discovery rate and multiple comparisons corrections, and reached statistical significance (*p* < 0.05). (**a**) Metabolically healthy obese (MHO; *n* = 10) subcutaneous (SQ) adipose tissue (AT) pathway increases compared to metabolically healthy lean (MHL; *n* = 12). (**b**) Metabolically unhealthy obese (MUO, *n* = 13) SQ AT pathway increases and decreases compared to MHL (*n* = 12). (**c**) Heat map of differentially expressed genes in MHO SQ AT compared to MUO (MUO *n* = 13; MHO *n* = 10). Specific transcripts are not denoted in this heat map.

We performed bulk RNA-seq on SQ AT biopsies to identify transcript expression differences between groups. In total, 15,423 transcripts were identified after the removal of non-protein coding genes and genes ambiguously mapped to chromosomes. The largest number of genes that were differentially expressed was between MHO and MHL AT (*n* = 1092), whereas only 56 genes were differentially expressed between the MHO and MUO groups, 154 genes were differentially expressed between the MHL and MUL groups, and 347 were differentially expressed between the MUL and MUO groups. MHO animals demonstrated upregulation of transcripts related to lipid synthesis, lipid dynamics and lipid transport and downregulation of transcripts related to inflammation initiation (Figure 3a,b), while MUO animals demonstrated the inverse pattern (Table 2 and Table 3; Supplementary Figure S5). Gene ontology term analysis revealed gene pathways specific to healthy obesity. Both the “acyl-CoA dehydrogenase activity” and “flavin adenine dinucleotide binding” pathways were upregulated in the MHO compared to the MHL, indicating that MHO animals upregulate transcripts related to fatty acid oxidation (Figure 3a). MHO animals also illustrated enrichment of the “metabolic process” pathway. Importantly, no immune response-related pathways were found to be differently enriched between the MHO and MHL groups. On the contrary, MUO animals illustrated downregulation of the “fatty acid metabolic process”, “acyl-CoA biosynthetic process”, and “lipid metabolic process” pathways compared to MHL (Figure 3b). MUO animals demonstrated increases in “immune response” and “cell chemotaxis” pathways compared to MHL, a signature not seen in MHO SQ adipose (Figure 3b). The “immune response” gene ontology term is comprised of 1973 genes, including those related to the innate immune response, B and T cell receptor signaling, and macrophage cytokine production. The “cell chemotaxis” gene ontology term is comprised on 316 genes, including those related to lymphocyte and T cell chemotaxis. Specific genes in these terms that were upregulated in the MUO compared to the MHL SQ AT included C-X-C Motif Chemokine Ligand 16 (*CXCL16*), C-C Motif Chemokine Ligand 11 (*CCL11*), *CCL22*, and *CCL26*. Pathway analysis between MUL and MUO groups identified enrichment of immune regulating pathways with combined obesity and metabolic disease (Supplementary Figure S4). MUO AT demonstrated significant downregulation of the “cellular respiration” and “tricarboxylic acid cycle” gene ontology pathways compared to MHL. This evidence of decreased mitochondrial function in the MUO but not in MHO aligns with our previous study where we identified poor mitochondrial quality control markers in MUO compared to MHO NHPs [15]. Gene term enrichment identified from comparisons of MHO and MUO, and MHL and MUL animals did not survive corrections. While they were ultimately not statistically significant, the terms “extracellular region”, “serine-type endopeptidase activity” and “transmembrane activity” were enriched in the MHO compared to the MUO (Supplementary Figure S5).

Transcripts upregulated in MHO included fatty acid binding protein 7 (*FABP7*), monoacylglycerol O-acyltransferase 1 (*MOGAT1*), ATPase phospholipid transporting 8B3 (*ATP8B3*), glycerol-3-phosphate acyltransferase 3 (*GPAT3*), and adrenoceptor beta 3 (*ADRB3*). Upregulation of *FABP7* indicates that MHO animals have improved signaling for fatty acid movement across membranes (Table 2). Downregulation of C-C motif chemokine receptor 1 (*CCR1*), pancreatic lipase related protein 1 (*PNLIP*), macrophage scavenger receptor 1 (*MSR1*), and oxidized low-density lipoprotein receptor 1 (*OLR1*) was observed. Activation of *MSR1* has been related to a phenotypic macrophage switch from anti-inflammatory to pro-inflammatory [16]. Therefore, downregulation of *MSR1* in MHO adipose indicates maintenance of an anti-inflammatory M2-based macrophage milieu, which coincides with the macrophage data presented above. The MHO animals’ lower expression of *CCR1, PNLIP,* and *OLR1* also suggests decreased tissue inflammation, atherogenic capacity and lectin activation [17,18]. The sustained anti-inflammatory environment in the MHO adipose occurred in the absence of differences in adipocyte size when compared to the MUO (Figure 1e) and radiographic adipose or SQ AT cell density (Table 1) differences.

In contrast, MUO AT demonstrated upregulated expression of transcripts related to inflammatory processes and glycogen storage (Table 3), including plasminogen (*PLG*) and glycogen synthase 2 (*GYS2*). Heats maps of differentially expressed genes between the two obese groups and the MHL group are shown in Supplementary Figure S6. Though inverse transcript expression was observed between MHO and MUO groups (Figure 3c), no differentially expressed transcripts between these groups met corrections for multiple comparisons. While they did not meet statistical inquiry, transcripts associated with inflammation, including *CCL19* and *CCL22*, were downregulated in MHO AT compared to MUO, corroborating previous findings [19]. Transcripts associated with nutrient transport, including solute carrier family *(SLC) 22A3* and *SLC10A6*, were upregulated in MHO AT compared to MUO (Supplementary Table S3) supporting an expression pattern consistent with low inflammation and high lipogenic capacity.

In line with these transcript expression differences between groups, the SQ AT M2/M1 ratio positively correlated with expression levels of ATP binding cassette subfamily C member 2 (*ABCC2*) (r = 0.315, *p* = 0.0448), and negatively correlated with expression levels of cyclin dependent kinase inhibitor 1A (*CDKN1A*) (r = −0.419, *p* = 0.00646), immunoglobulin heavy constant delta (*IGHD*) (r = −0.398, *p* = 0.00988), and heat shock transcription factor 4 (*HSF4*) (r = −0.436, *p* = 0.00436). These associations indicate that increases in the M2/M1 ratio correspond with decreased antigen binding, cellular senescence, and stress response, and improved movement of molecules across membranes (Supplementary Table S4), and support the MHO-specific downregulation of inflammatory transcripts and upregulation of lipid handling-related transcripts noted above.

### 2.4. Tissue Cytokine Burdens Match Tissue Macrophage Profiles

We found no group differences in circulating adiponectin, MCP-1, IL-6, IL-1β or PAI-1 (Supplementary Table S5), but a number of biologically relevant relationships were present. Circulating levels of PAI-1 demonstrated a trend toward a positive association with the increasing percentages of M1 macrophages (r = 0.27, *p* = 0.09). Circulating levels of PAI-1 also were negatively correlated with percentages of M2 macrophages (r = −0.29, *p* = 0.06), indicating that pro-inflammatory PAI-1 levels decrease as anti-inflammatory macrophage densities increase. Tissue levels of MCP-1 differed by obesity status (*p* = 0.02), where obese animals displayed higher levels. Tissue MCP-1 also demonstrated a trend toward significance by health status (*p* = 0.06), where unhealthy animals had higher levels (Supplementary Table S5). SQ AT levels of anti-inflammatory IL-10 were significantly different between groups (*p* = 0.03), where the MHL group had the highest detectable tissue levels (Supplementary Table S4). Tissue levels of IL-10 also differed significantly by obesity status (*p* = 0.004), where obese animals demonstrated significantly lower levels (Supplementary Table S5). Tissue levels of TGF-β exhibited (1) a trend toward significance (*p* = 0.09), where the MUL group displayed increased tissue levels, and (2) a trend toward a significant difference by health, where unhealthy animals illustrated higher tissue levels (*p* = 0.08). Additionally, abdominal SQ AT concentrations of TGF-β negatively correlated with the SQ AT M2/M1 macrophage ratio (r = −0.351, *p* = 0.045). No other cytokines measured correlated with the M2/M1 macrophage ratio. Accordingly, our tissue cytokines tracked with our observed macrophage phenotypic shifts.

### 2.5. Changes in Tissue Vasculature or Fibrosis Do Not Explain Poor Health but Relate to Adipocyte Hypertrophy

**Figure 4:**
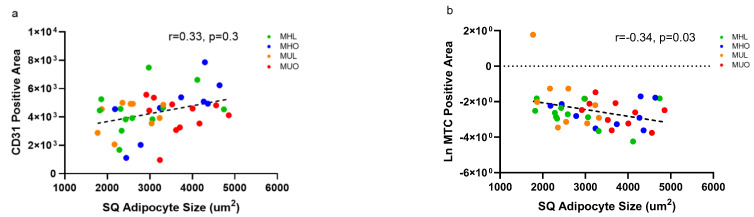
Endothelial area increases with increasing subcutaneous adipose adipocyte size, while fibrosis decreases with increasing subcutaneous adipose adipocyte size. (**a**) Correlation between CD31-positive staining area and SQ adipocyte size (Pearson’s r = 0.33, *p* = 0.03, *n* = 43). (**b**) Correlation between transformed percent MTC-positive area and SQ adipocyte area (Pearson’s r = −0.34, *p* = 0.03, *n* = 43).

Hematoxylin and eosin staining was used to evaluate adipocyte size differences, while CD31 staining and Masson’s Trichrome staining (MTC) were used to quantitate tissue vasculature and fibrosis, respectively (Supplementary Figure S7). No differences in either CD31-positive area or percent MTC-positive staining were present between groups (Supplementary Table S6). CD31-positive area correlated positively with adipocyte size (r = 0.33, *p* = 0.03; Figure 4a), indicating that as adipocyte size increased, the vasculature increased, possibly to meet adipocyte nutrient requirements. Percent MTC-positive staining correlated negatively with increasing SQ adipocyte size (r = −0.34, *p* = 0.03; Figure 4b), highlighting that as adipocyte size increased, interstitial collagen I formation decreased. Additionally, no expression differences in other ECM transcripts, including collagen types V, VI, VII and IX, were observed in MHO AT. Collagens can be upregulated in aberrant obesity [20]. Our MUO SQ AT demonstrated upregulation of *COL4A3* and downregulation of *MMP7* compared to the MHL indicating deposition of fibers with decreased breakdown (Table 3). No other ECM transcripts, including other matrix metalloproteinases, laminins, or fibronectins, were differentially expressed between groups. Our results indicate that neither tissue fibrosis nor vasculature changes explain health status in lean or obese states. Rather, we posit that metabolic dysfunction in the presence and absence of obesity may be caused by other factors, such as the differences in immune cell populations and adipocyte function shifts observed here.

## 3. Discussion

In this study, we identify features of naturally occurring obesity in primates, with the unique determination that upregulation of lipid handling-related transcripts and an increased M2/M1 macrophage ratio are associated with maintained health despite obesity. Unhealthy obesity was characterized by an opposing profile with a decrease in the SQ AT M2/M1 ratio, tissue immune activation and metabolic process downregulation. Previous characterization of metabolically healthy obese individuals determined that maintained health with obesity corresponded with upregulated mitochondrial oxidative phosphorylation and fatty acid oxidation transcript expression, decreased levels of markers of chronic inflammation, and reduced liver adipose accumulation [21]. Additionally, expression of *SLC39A8* and *SLC16A7*, solute carrier family members, in circulation or saliva were associated with maintained metabolic health in obese women [22,23]. Our work verifies these findings and adds vital information regarding differences in local immune cell populations in healthy and unhealthy obesity. Supporting our SQ AT immune cell findings, reductions in the M2/M1 AT macrophage ratio previously correlated with increasing insulin resistance [24,25]. In our study, the M2/M1 ratio negatively correlated with expression levels of transcripts related to antigen binding, stress response, and cellular senescence, providing further evidence that an increase in the M2/M1 macrophage ratio is associated with improved local health. Additionally, human adipose spatial mapping indicates that the M2 macrophage subtype is the predominant macrophage [26] and, thus, a more important macrophage phenotype than has been appreciated to date. Even more surprising and perhaps importantly, healthy obesity correlated with higher M2 macrophage levels than seen even in their healthy lean counterparts.

Our observed macrophage and transcriptional shifts occurred without changes in abdominal adipose distribution or ectopic adipose accumulation. Western dietary components redistribute adipose to ectopic sites [27,28] and drive increases in pro-inflammatory MCP-1 and AT macrophages [29,30,31]. Our data were derived from nonhuman primates unexposed to an unhealthy diet. Though the animals consumed a healthy low-fat, low-sugar, high-fiber, and high-protein chow, we recognize that our cohort animals illustrated more abdominal VIS AT accumulation than SQ AT accumulation, which resulted in VIS:SQ ratios over 1. Previous work has shown that human females without cardiovascular disease have an abdominal VIS:SQ AT ratio under 1 [32]. While our animal model demonstrates a higher abdominal VIS:SQ AT ratio, they present the full range of metabolic abnormalities that are present in people, and they demonstrate the same MHO to MUO transition rate [33]. Additionally, our obese animals demonstrated lower VIS:SQ ratios as a result of increased SQ AT expansion with obesity onset. This corresponds with the animals’ consumption of a healthy diet and their healthy environment, and contributed to the premise of this work, which was to evaluate the differing properties of SQ AT as it expands.

Our data suggest that driving AT macrophage polarization toward an anti-inflammatory M2 profile and enhancing AT fatty acid oxidation may improve or maintain tissue health with obesity onset. MHL and MUL SQ AT demonstrated increased percentages of the undefined macrophages compared to obese animals, indicating that, in the absence of obesity and poor diet consumption, fewer inflammatory signals result in reduced macrophage polarization. In line with this, while the MHL SQ AT demonstrated a non-significant increase in intermediate macrophages, no increases in MHL SQ AT pro-inflammatory cytokine protein expression or increases in inflammation-related transcript expression were observed. Current treatment options for MUO persons rest primarily on weight loss. Our findings support the implementation of lifestyle strategies, such as healthy diets and exercise training, which have been shown to induce M2 macrophage polarization and improve AT function [34], recapitulating the MHO AT state without the need for weight reduction. AT mesenchymal stem cells regulate the tissue immune response through exosomes, as stem cells cluster near M2 macrophages and drive additional M2 polarization [26,35]. Stem cell-derived exosomes from healthy adipose were capable of inducing M2 AT macrophage polarization, highlighting a promising future direction for the treatment of MUO persons [36]. Additionally, treatment with bezafibrate induced acyl-CoA dehydrogenase in cultured fibroblasts while decreasing *TNF-α* [37], replicating an MHO-like physiologic state, where fatty acid degradation is increased. Along these lines, recent work identified that the use of a near-infrared fluorophore (IR-61) increases AT macrophage oxidative phosphorylation and reduces inflammation, and could be a useful agent for the treatment of obesity-related metabolic dysfunction [38].

Long-term follow-up on MHO patients determined that half of MHO individuals do not develop metabolic abnormalities within ten years [33]. Our findings validate the MHO classification and highlight differences in a key endocrine tissue that contributes to maintaining health with obesity. We used a nonhuman primate model that naturally and reliably demonstrates the four health groups and a strict definition of metabolically healthy versus unhealthy obesity, in contrast to studies which have classified MHO people and animals as obese with an additional metabolic derangement [39]. Nonhuman primates are the leading research model in this arena as, in addition to demonstrating health group distributions that are similar to people, their adipose tissue accumulation patterns [40], immune cell profiles [41], and mechanisms of lipolytic control [42] closely match those observed in humans. While the animals in this study were all female and hormones, including 17-β-estradiol, can protect against metabolic derangements [43], the selected NHP model demonstrated distinct health profiles with significant differences in MetS risk factors, which categorized them into the four clinically defined metabolic health groups.

We immunohistologically evaluated the most predominant immune cell type in SQ white adipose but acknowledge that there are significant numbers of B and T cells that reside in and play key immune modulatory and/or functional roles in maintaining metabolic homeostasis in AT [44,45]. An immunohistochemical approach was chosen, as it has been shown to identify more in situ macrophages without disrupting the tissue inflammatory state compared to other methodologies [46]. Additionally, our staining strategy allowed us to capture a spectrum of macrophage subtypes. We used positive co-staining of both CD68 and CD163 to identify all macrophages, as CD163 has been identified as being present in both resident and recruited macrophages, and CD163 cells have been noted to be increased in both healthy and unhealthy adipose [5,47]. We elected to use the transcription factors pSTAT1 and cMAF as co-stains for M1 and M2 macrophages, respectively. M1 macrophage polarization is promoted after IFN-γ/signal transducer and activator of transcription 1 (STAT1) pathway activation [48]. Similarly, cMAF acts as a metabolic checkpoint regulating the TCA cycle and UDP-GlcNAc biosynthesis, promoting M2 macrophage polarization [49,50], compared to CD206, which is a surface marker of both dendritic cells and macrophages and, thus, less specific [51].

We coupled our immunohistochemical approach with tissue cytokine measurements to secondarily assess inflammatory markers associated with macrophage types, and unbiased RNA-seq supported our inflammation-related findings. While this work was underpowered to detect significant differences in tissue cytokines, the trends observed corroborate the significant tissue immune cell profiles, and future work will include increased sample sizes. In line with this, obese animals demonstrated decreases in SQ AT IL-10 compared to lean animals as the obese animals displayed increased macrophage polarization, or response to inflammatory signaling. Specifically, MHL and MUL SQ AT showed increases in undefined macrophages while MHO SQ AT illustrated increases in M2 macrophage polarization. While RNA-seq does not allow for cell type specificity, it provides accurate gene expression quantification without requiring qPCR validation, as strong correlations have been observed between the two methods [52]. Our histological approaches concluded that there was comparable tissue vascularity but could not assess nutritive perfusion, though *HIF1a* and related genes were not differentially expressed, suggesting that gross hypoxia was not driving AT differences. Similarly, fibrosis by MTC staining identifies predominantly collagen I. Fibrosis and related gene transcripts were not related to health differences nor MHO M2 macrophage enrichment. However, changes in MUO AT genes, including upregulation of *COL4A3* and downregulation of *MMP7*, may indicate dysregulation as previously described [53].

The strengths of our study include the peerless animal model, which allows MHO AT character to be explored when body adipose distributions, adipocyte size and macrophage density are all comparable. Thus, we report that a unique MHO SQ AT transcript expression profile suggests that a preferred adipocyte function co-exists with a specific immune cell profile. In conclusion, we identify M2 macrophages as an SQ AT component that corresponds with sustained metabolic health in the obese state and a novel target for improving health under such conditions.

## 4. Methods and Materials

### 4.1. Study Design and Cohort Selection

A 44-animal age-matched cohort was selected from the Vervet Research Colony (VRC) at Wake Forest University School of Medicine (WFSM). We used a modified version of the National Cholesterol Education Program (NCEP) ATP III definition [54] to categorize our animals, as these colony-raised monkeys exclusively consume a healthy low-fat, low-cholesterol and low-sugar diet and live in a controlled indoor/outdoor setting for the entirety of their lives. We used metabolic syndrome (MetS) criteria, with criteria including the waist measure adjusted for NHPs (waist circumference ≥ 40 cm), HbA1c > 6, fasting glucose ≥ 100 mg/dL, systolic blood pressure > 135 mmHg, diastolic blood pressure > 85 mmHg, triglycerides ≥ 125 mg/dL, HDL cholesterol ≤ 50 mg/dL to identify animals in each health group [15]. MetS scores were calculated as the sum of each MetS criterion met [15]. MHL animals had a MetS score of zero, MHO animals had a MetS score of one for their obesity status only, and MUL and MUO animals had MetS scores of two or higher. We have previously described that African green monkeys demonstrate metabolic health group distributions and MetS risk factors similar to people [15,55,56]. MetS in people is typically defined as the presence of three or more MetS risk factors. However, we found the percentage of African green monkeys with two or more metabolic syndrome criteria to be 29%, which aligns with the percentage of Americans identified as having MetS [57] and formed the basis of our groupings. Given that our animals are born and housed in such healthy environments, we found that 36% of screened African green monkeys demonstrate zero MetS risk factors [15], which is three-fold higher than the prevalence of American adults without MetS risk [56]. Accordingly, a MetS score of two or higher indicates overt unhealthiness in this animal model.

Waist circumference determined obesity. A waist circumference of ≥40 cm corresponds with the upper 20th percentile of the VRC animals [55]. This percentile cutoff was modeled according to where the Adult Treatment Panel III risk waist definition falls for males and females in normal human populations [55,58,59,60]. A waist circumference of 40 cm represents mild obesity, while a waist circumference of ≥44.5 cm represents severe obesity and the upper 10th percentile of the VRC animals. Three animals in each obese group were classified as severely obese. Groups were selected to balance their relatedness coefficients (Supplementary Table S7) to avoid family overrepresentation in any phenotypic group. All animals were socially housed and consumed a low-fat, low-cholesterol, high protein and fiber chow with no added sugar (Lab Diet 5038; LabDiet).

We used the animals’ group placements at the time of biopsy when conducting our analyses, allowing for 9–13 observations per group for each endpoint. As with many NHP studies, selection from the breeding colony led to our study including only females.

All procedures were performed in accordance with the Guide for Care and Use of Laboratory Animals. Protocols for the avoidance of pain and discomfort were adhered to and conducted in compliance with the WFSM Institutional Animal Care and Use Committee.

### 4.2. Blood and Metabolic Measures

Animals were fasted overnight and anesthetized with intramuscular ketamine injections (10–15 mg/kg). Animals were weighed and had waist circumference measured with a flexible tape measure at the level of the umbilicus. Blood samples were obtained via percutaneous femoral venipuncture into ethylenediaminetetraacetic acid (EDTA) blood tubes and placed on ice until processed for plasma, which was stored at −80 °C until analyzed. Fasting glucose was determined by the glucose oxidase method, and fasting insulin concentrations were determined by enzyme-linked immunosorbent assay performed using isolated plasma (ELISA; Mercodia, Winston-Salem, NC, USA; cat. no. 10-1113-01). Whole blood was used to measure glycated hemoglobin using high-performance liquid chromatography methodology (Afinion Alere, Abbott, Chicago, IL, USA). Triglyceride, high-density lipoprotein cholesterol (HDLc), circulating free fatty acid (Abcam, Cambridge, UK; cat. no. ab65341), and total cholesterol concentrations were measured enzymatically using isolated plasma [61]. Blood pressure was measured indirectly by sphygmomanometer as previously described [61]. Supplementary Figure S8 illustrations the methods performed on collected blood samples and isolated plasma.

### 4.3. Body Composition

Computed tomography (CT) scans were performed on a GE 0.625-mm slice Discovery MI DR scanner (GE Healthcare, Chicago, IL, USA). Images were reconstructed with TeraRecon Aquarius Intuition software (Durham, NC, USA) and converted into dicom format for analysis. Fat- and lean-containing voxels were used to calculate volume results and expressed as a percentage of the animal’s total body weight [62]. Abdominal fat distribution was assessed by regional analysis between the thoracolumbar junction and the fourth lumbar vertebrae using automated differentiation of deposits within and outside the body wall [62]. Liver attenuation values were collected in triplicate [62], where liver attenuation, or density, was used to determine liver adipose accumulation.

### 4.4. Tissue Collections

Animals under isoflurane anesthesia had SQ AT collected from the ventral aspect of the caudal abdomen at the umbilicus. Upon collection, tissues were washed twice in Dulbecco’s phosphate-buffered saline (DPBS) and either flash frozen in liquid nitrogen or placed in 4% paraformaldehyde (PFA). Supplementary Figure S8 depicts the methods performed on collected adipose tissues.

### 4.5. Cytokine Evaluations

Protein was extracted from ~100 mg of frozen tissue using the Invent Minute Total Protein Extraction Kit for Adipose Tissues (Plymouth, MN, USA; cat. no. AT-022) according to the manufacturer’s instructions. Extracted protein was quantitated by bicinchoninic acid assay. A total of 75 μg of protein was used to analyze monocyte chemoattractant protein-1 (MCP-1), transforming growth factor beta (TGF-β), interleukin (IL)-6, (R & D Systems, Minneapolis, MN, USA; cat. nos. DCP00, DB100B, and D6050) and IL-10 by ELISA (U-Cytech Biosciences, Utrecht, The Netherlands; cat. no. CT146). Frozen plasma samples were used to evaluate circulating MCP-1, IL-6, interleukin (IL)-1β (cat. no. DLB50), plasminogen activator inhibitor (PAI)-1 (cat. no. DSE100), and adiponectin (cat. no. DRP300) via ELISA (R & D Systems, Minneapolis, MN, USA). Assays were run in triplicate using predetermined dilutions.

### 4.6. Histology and Immunohistochemistry

PFA-fixed, paraffin-embedded tissues were used to create histologic sections. Sections were stained with fluorescent CD68 and CD163 to evaluate total macrophage infiltration (AbD Serotec Bio-Rad, Hercules, CA, USA; 1 μg/mL), and co-stained with pSTAT1 (Cell Signaling Technology, Danvers, MA, USA, cat. no. 9167S; 1:250 dilution) and CMAF (Abcam, Cambridge, UK; cat. no. ab199424; 1:250 dilution) identify undefined, M1, intermediate, and M2 macrophages [47]. Undefined macrophages were identified as CD68^+^CD163^+^, M1s were identified as CD68^+^CD163^+^pSTAT1^+^, intermediate macrophages were identified as CD68^+^CD163^+^pSTAT1^+^CMAF^+^, and M2s were identified as CD68^+^CD163^+^CMAF^+^. A Visiopharm software macro (v.2019.02.1.6005) distinguished between macrophage types. Macrophage subtypes were identified across an entire tissue section. Macrophage subtype percentages were determined by taking the number of the specific macrophage subtype and dividing by the total number of macrophages identified in a tissue section. Macrophage density was determined by taking the total number of macrophages for a tissue section and dividing by the tissue area, which was calculated using a specialized Visiopharm macro. Unstained sections and lung tissue were used as negative and positive controls, respectively. Adipocyte analyses were performed using a Visiopharm (v.2019.02.1.6005) macro on hemoxylin and eosin-stained slides that determined total tissue area, total adipocyte number, total adipocyte area, and individual adipocyte area. Adipose sections were stained with Masson’s Trichrome (Abcam, Cambridge, UK) and CD31 (Biocare Medical, Pacheco, CA, USA; cat. no. CM131C) and evaluated using Visiopharm.

### 4.7. Transcriptomic Analyses

RNA was extracted from 100mg adipose biopsies (*n* = 43) using the Qiagen RNeasy Lipid Mini tissue kit (Germantown, MD, USA; cat. no. 74804) according to the manufacturer’s protocol. Genomic DNA was removed using the Qiagen RNase-Free DNase set according to the manufacturer’s protocol. Total RNA was used to prepare cDNA libraries using the Illumina^®^ TruSeq Stranded mRNA Library Prep and IDT for Illumina-TruSeq RNA UD Indexes. RNA quality was assessed using the Agilent TapeStation 4150. RIN values ranged from 7.2 to 9.2. Poly-A selection using oligo-dT magnetic beads occurred and was followed by enzymatic fragmentation, reverse-transcription and double-stranded cDNA purification using AMPure XP magnetic beads. The cDNA was end repaired, 3′ adenylated with Illumina sequencing adaptors ligated onto the fragment ends, and the stranded libraries were pre-amplified with PCR. The library size distribution was validated and quality inspected using an Agilent 2100 Bioanalyzer. The quantity of each cDNA library was measured using the Qubit 3.0 (Thermo Fisher, Waltham, MA, USA). The libraries were pooled and sequenced to a target read depth of 25M reads per library using single-end, NovaSeq 6000 SP Reagent Kit (100 cycles) (Illumina Inc., San Diego, CA, USA) on the NovaSeq 6000. RNA-seq data were screened using FastQC for quality analysis. The STAR sequence aligner and the vervet monkey genome (*Chlorocebus aethiops)* were used to align clean reads [63]. 32.52 ± 5.16 million reads were uniquely mapped. “featureCounts” quantified RNA-seq data as counts [64]. Differentially expressed genes (DEGs) were analyzed using the Limma-voom tool in R, where gene counts were used as inputs [65]. Database for Annotation, Visualization and Integrated Discovery (DAVID) was to identify biologically enriched gene ontology (GO) terms (https://david.ncifcrf.gov/) (accessed on 1 November 2021).

### 4.8. Statistical Methods

The Shapiro–Wilk and Levene’s tests were used to assess normality and homogeneity of variance for histological and protein endpoints. Adipocytes under 400 um^2^ in size were removed prior to downstream analyses. Endpoints that did not meet assumptions underwent logarithmic transformation. Analysis of variance was performed on all histological and protein endpoints to determine group differences. Factorial analysis of variance determined the main effects of obesity and health status. Pearson’s correlation was used to determine associations. A *p*-value of <0.05 was used to determine significance and a *p*-value of <0.1 was used to determine a trend. Significant DEGs were defined as *p* < 0.01 and log2 fold change > |0.585| (1.5-fold change).

## Figures and Tables

**Table 1:** Nonhuman primate cohort demographic information. Values are presented as means with SEM in parentheses. ANOVA and factorial ANOVA were used to identify differences between groups and by health and obesity status. A total of 44 female African green monkeys were selected for investigation. Metabolically healthy lean (MHL; *n* = 12); metabolically healthy obese (MHO; *n* = 10); metabolically unhealthy lean (MUL; *n* = 9); metabolically unhealthy obese (MUO; *n* = 13).

Measurement	MHL	MHO	MUL	MUO	ANOVA *p*-Value	Health *p*-Value	Obesity *p*-Value
Age (yrs)	15.15 (1.30)	15.14 (1.55)	14.32 (1.99)	15.87 (0.92)	0.74	0.69	0.26
Body Weight (kg)	5.25 (0.26)	6.47 (0.24)	4.95 (0.24)	6.91 (0.26)	<0.001	0.19	0.01
Waist Circumference (cm)	34.29 (0.97)	42.86 (1.07)	34.80 (0.78)	43.23 (0.85)	<0.001	0.02	<0.001
Fasting Glucose (mg/dL)	73.5 (3.20)	67.2 (3.59)	108.67 (19.15)	104.32 (12.37)	0.01	0.36	0.86
Glycosylated Hemoglobin A1c (%)	4.07 (0.05)	4.07 (0.07)	4.83 (0.58)	5.38 (0.57)	0.009	0.012	0.31
Systolic Blood Pressure (mmHg)	114.44 (3.79)	110.10 (5.43)	144.78 (4.95)	111.62 (5.06)	<0.001	0.48	0.31
Diastolic Blood Pressure (mmHg)	67.53 (3.38)	61.57 (3.80)	89.22 (4.70)	66.79 (2.46)	<0.001	0.25	0.42
High Density Lipoprotein Cholesterol (mg/dL)	68.08 (3.37)	56.10 (5.76)	73.44 (10.38)	62.50 (4.39)	0.24	0.33	0.06
Triglycerides (mg/dL)	56.34 (3.46)	55.90 (5.89)	79.10 (15.67)	90.52 (10.80)	0.03	0.24	0.76
Liver Density (HU)	60.37 (2.26)	55.71 (4.03)	59.54 (2.84)	57.59 (1.94)	0.86	0.69	0.83
% Fat Mass	18.35 (2.69)	36.59 (2.81)	16.70 (2.07)	35.98 (3.05)	0.01	0.94	0.06
% Lean Mass	54.13 (1.56)	47.29 (2.89)	55.03 (1.52)	45.63 (1.60)	0.02	0.91	0.07
Visceral:Subcutaneous Adipose Area (cm^2^)	3.44 (0.46)	1.78 (0.19)	3.86 (0.83)	1.73 (0.30)	0.31	0.88	0.19
Plasma Insulin (mU/L)	16.80 (4.15)	19.98 (3.87)	8.84 (2.08)	51.12 (11.18)	0.0055	0.71	0.0019

**Table 2:** Top false discovery rate correction-surviving up- and downregulated transcripts illustrated in the metabolically healthy obese (*n* = 10) subcutaneous adipose tissue compared to the metabolically healthy lean (*n* = 12).

Transcript	Log Fold Change	Adjusted *p*-Value
Fatty Acid Binding Protein 7 (*FABP7*)	1.51	0.010
Prostaglandin D2 Synthase (*PTGDS*)	1.53	0.015
Aldehyde Dehydrogenase 1 Family Member B1 (*ALDH1B1*)	1.56	0.044
Transferrin (*TF*)	1.66	0.024
Thyroid Stimulating hormone receptor (*TSHR*)	1.70	0.0036
Lactate Dehydrogenase D (*LDHD*)	1.74	0.00016
Phosphoenolpyruvate Carboxykinase 2 (*PCK2*)	1.77	0.0063
ATPase Phospholipid Transporting 8B3 (*ATP8B3*)	1.79	0.0063
Adrenoceptor Beta 3 (*ADRB3*)	1.79	0.010
Trefoil Factor 3 (*TFF3*)	1.88	0.027
ATP Binding Cassette Subfamily C Member 6 (*ABCC6*)	1.89	0.0051
Phosphoenolpyruvate Carboxykinase 1 (*PCK1*)	2.05	0.0075
Monoacylglycerol O-Acyltransferase 1 (*MOGAT1*)	2.12	0.00071
Glycerol-3-Phosphate Acyltransferase 3 (*GPAT3*)	2.16	0.015
Protein Phosphatase 1 Regulatory Inhibitor Subunit 1B (*PPP1R1B*)	2.38	0.034
ADAM-Like Decysin 1 (*ADAMDEC1*)	−4.11	0.00011
Cartilage Oligomeric Matrix Protein (*COMP*)	−3.13	0.00022
Sterile Alpha Motif Domain Containing 5 (*SAMD5*)	−2.98	0.000056
Dendrocyte Expressed Seven Transmembrane Protein (*DCSTAMP*)	−2.93	0.014
R-Spondin 1 (*RSPO1*)	−2.76	0.0034
Thymic Stromal Lymphopoietin (*TSLP*)	−2.52	0.00067
Osteoclast Stimulatory Transmembrane Protein (*OCSTAMP*)	−2.46	0.045
CUB and Sushi Multiple Domains 2 (*CSMD2*)	−2.31	0.011
ETS Homologous Factor (*EHF*)	−2.28	0.027
Oxidized Low Density Lipoprotein Receptor 1 (*OLR1*)	−2.23	0.0063
Carboxypeptidase Z (*CPZ*)	−2.20	0.029
C-C Motif Chemokine Receptor 1 (*CCR1*)	−2.17	0.00070
Serpin Family A Member 5 (*SERPINA5*)	−2.11	0.0020
Pancreatic Lipase Related Protein 1 (*PNLIPRP1*)	−2.10	0.020
Macrophage Scavenger Receptor 1 (*MSR1*)	−2.08	0.014

**Table 3:** Top false discovery rate correction-surviving up- and downregulated transcripts in metabolically unhealthy obese (*n* = 13) subcutaneous adipose tissue compared to metabolically healthy lean (*n* = 12).

Transcript	Log Fold Change	Adjusted *p*-Value
Glycogen Synthase 2 (*GYS2*)	3.72	0.0059
Collagen Type IV Alpha 3 Chain (*COL4A3*)	2.69	0.0084
Family with Sequence Similarity 131 Member C (*FAM131C*)	2.62	0.0067
Serum Amyloid A4, Constitutive (*SAA4*)	2.45	0.028
SH3 Domain Binding Kinase Family Member 2 (*SBK2*)	2.41	0.0011
Glycine-N-Acyltransferase (*GLYAT*)	2.40	0.00033
Plasminogen (*PLG*)	2.33	0.000012
Adrenoceptor Beta 3 (*ADRB3*)	2.28	0.0019
Stathmin 4 (*STMN4*)	2.23	0.0456
C-type Lectin Domain Family 4 Member G (*CLEC4G*)	2.22	0.0060
Thyroid Stimulating Hormone Receptor (*TSHR*)	2.21	0.000049
Monoacylglycerol O-Acyltransferase 1 (*MOGAT1*)	2.19	0.00013
Polypeptide N-Acetylgalactosaminyltransferase 9 (*GALNT9*)	2.17	0.0016
V-Set Furthermore, Transmembrane Domain Containing 2 Like (*VSTM2L*)	2.17	0.0043
Cat Eye Syndrome Chromosome Region, Candidate 2(*CECR2*)	2.11	0.0017
Glycoprotein Hormones, Alpha Polypeptide (*CGA*)	−2.62	0.0054
Osteoclast Stimulatory Transmembrane Protein (*OCSTAMP*)	−2.63	0.013
WAP Four-Disulfide Core Domain 2 (*WFDC2*)	−2.67	0.00025
Solute Carrier Family 5 Member 9 (*SLC5A9*)	−2.68	0.00038
Glutamate Ionotropic Receptor AMPA Type Subunit 1 (*GRIA1*)	−2.91	0.000011
Cadherin 1 (*CDH1*)	−2.98	0.0047
Sterile Alpha Motif Domain Containing 5 (*SAMD5*)	−2.99	0.000010
ETS Homologous Factor (*EHF*)	−3.00	0.00013
Dendrocyte Expressed Seven Transmembrane Protein (*DCSTAMP*)	−3.02	0.0040
WD Repeat Domain 64 (*WDR64*)	−3.12	0.0000085
Matrix Metallopeptidase 7 (*MMP7*)	−3.29	0.035
Solute Carrier Family 24 Member 2 (*SLC24A2*)	−3.32	0.00021
S100 Calcium Binding Protein A7A (*S100A7A*)	−3.32	0.00040
ADAM Like Decysin 1 (*ADAMDEC1*)	−4.06	0.000028
Death Associated Protein Like 1 (*DAPL1*)	−4.27	0.000056

## Data Availability

All transcriptomic data will be made available upon request.

## Supplementary Materials

The supporting information can be downloaded at: https://www.mdpi.com/article/10.3390/ijms232012680/s1.
