# Supplementary material for: Macrophage Phenotypes and Gene Expression Patterns Are Unique in Naturally Occurring Metabolically Healthy Obesity
Source: Int J Mol Sci. 2022 Oct 21;23(20):12680. doi: 10.3390/ijms232012680 (PMC9604193; doi:10.3390/ijms232012680)
Supplement: Supplementary file 1 [file ijms-23-12680-s001.zip › ijms-1910318-supplementary.pdf]

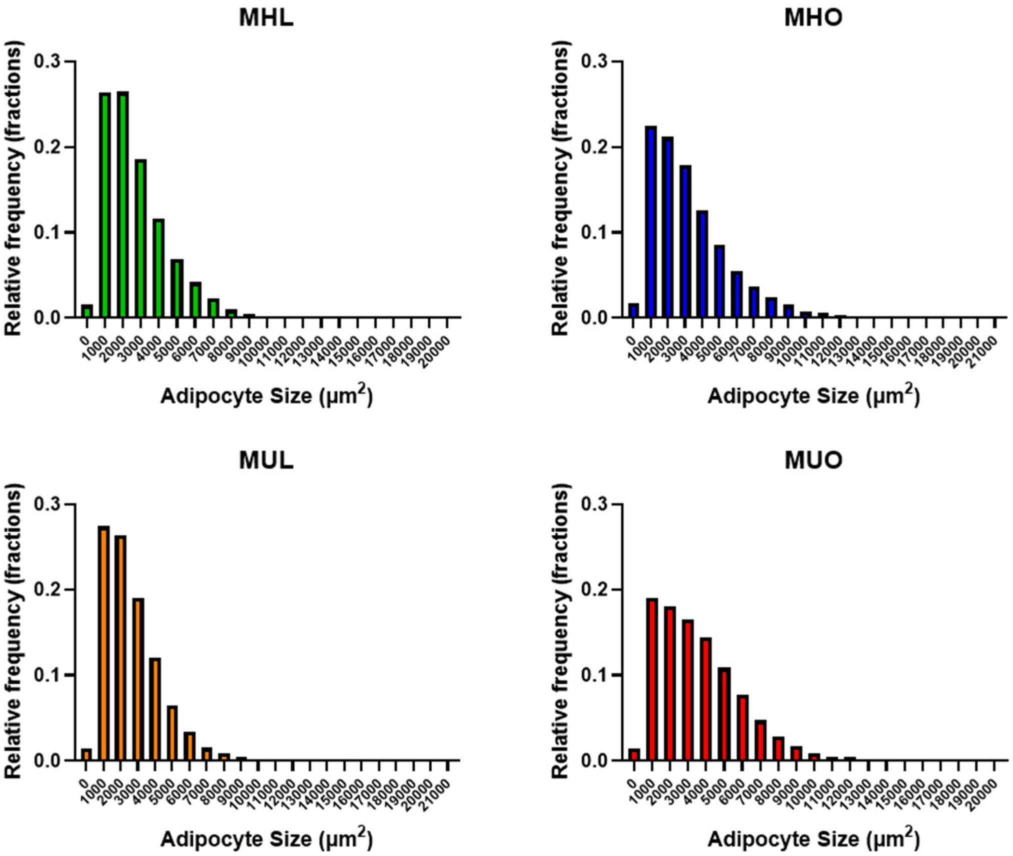

Supplementary Figure S1. Nonhuman primate subcutaneous adipose tissue adipocyte size frequency distributions. Adipocyte size frequency distributions for each metabolic health group. MHL = metabolically healthy lean (n=12); MHO = metabolically healthy obese (n=10); MUL = metabolically unhealthy lean (n=9); MUO = metabolically unhealthy obese (n=13).

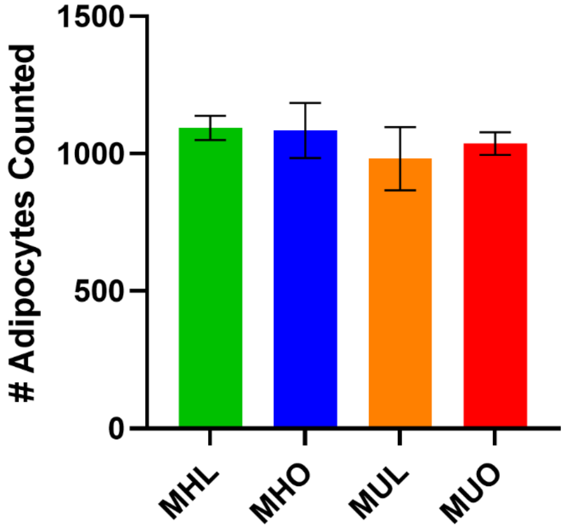

Supplementary Figure S2. Number of adipocytes evaluated. The total number of adipocytes counted for all animals in each group did not differ ($p>0.05$). MHL = metabolically healthy lean (n=12); MHO = metabolically healthy obese (n=10); MUL = metabolically unhealthy lean (n=9); MUO = metabolically unhealthy obese (n=13).

a

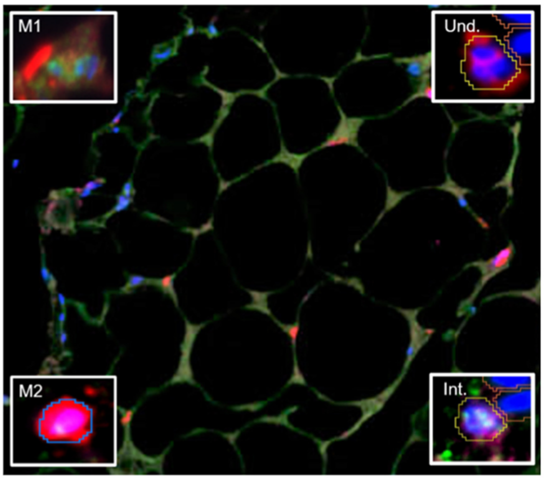

b

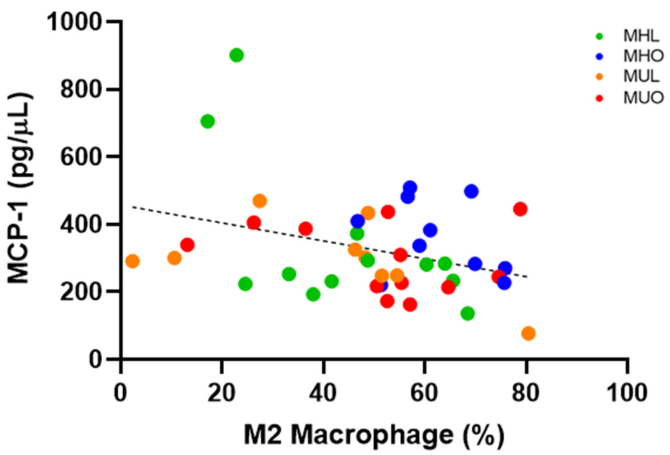

Supplementary Figure S3. Anti-inflammatory M2 adipose tissue (AT) macrophages negatively correlate with circulating monocyte chemoattractant protein (MCP)-1. (a) Example image of the immunofluorescent staining used to identify four macrophage subtypes. M1 macrophages were identified as CD163+CD8+pSTAT1+ and stained green. M2 macrophages were identified as CD163+CD68+CMAF+ and stained magenta. Undefined (Und) macrophages were identified as CD163+CD68+ and were stained red. Intermediate (Int) macrophages were identified as CD163+CD68+CMAF+pSTAT1+. Nuclei were stained with DAPI and appeared blue. The image was taken at 40x magnification. (b) A correlation analysis revealed that circulating monocyte chemoattractant protein (MCP)-1 is negatively associated with the percentage of M2 macrophages present in subcutaneous (SQ) adipose (r=-0.34, p=0.03, n=43). This association likely indicates that as more anti-inflammatory M2 macrophages inhabited the SQ adipose, secretion of pro-inflammatory MCP-1 decreased.

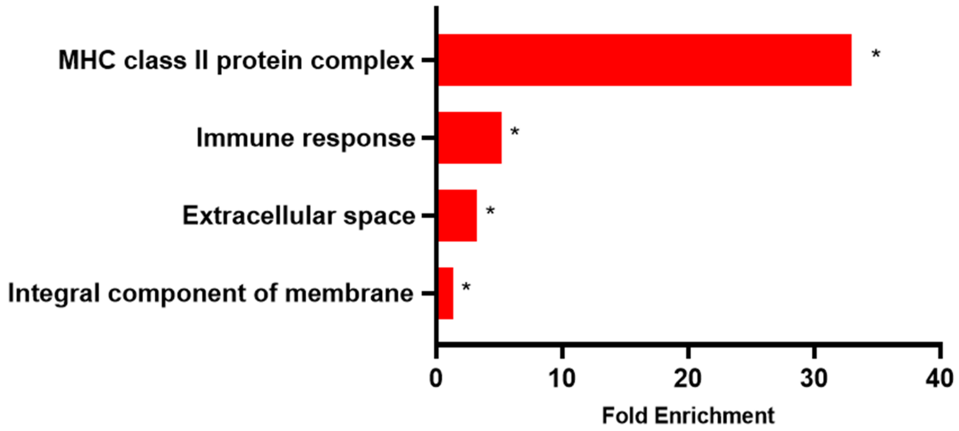

Supplementary Figure S4. Gene ontology term pathways enriched in metabolically unhealthy obese (MUO) subcutaneous adipose compared to metabolically unhealthy lean (MUL). The added effect of obesity on poor metabolic health results in upregulation of pathways related to immune response and expansion. Pathways displayed remained statistically significant (*=p<0.05) after false discovery rate and multiple comparisons corrections (MUL n=9; MUO n=13).

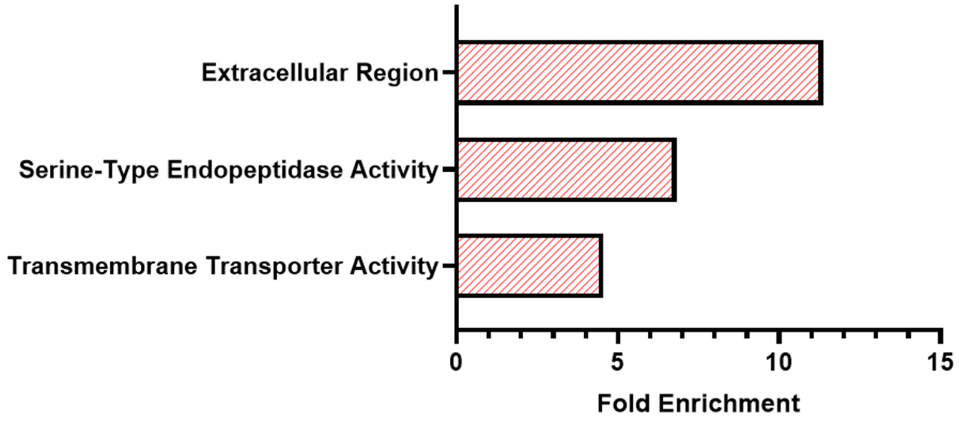

Supplementary Figure S5. Gene ontology term pathways enriched in the metabolically healthy obese (MHO) subcutaneous adipose compared to the metabolically unhealthy obese (MUO). Healthy obesity results in muted upregulation of term pathways associated with moving nutrients across membranes. However, the pathways shown did not survive false discovery rate and multiple comparisons corrections, so the data cannot be further interpreted. (MHO n=10; MUO n=13). Hatched bars indicate values that did not reach statistical significance (p>0.05).

a

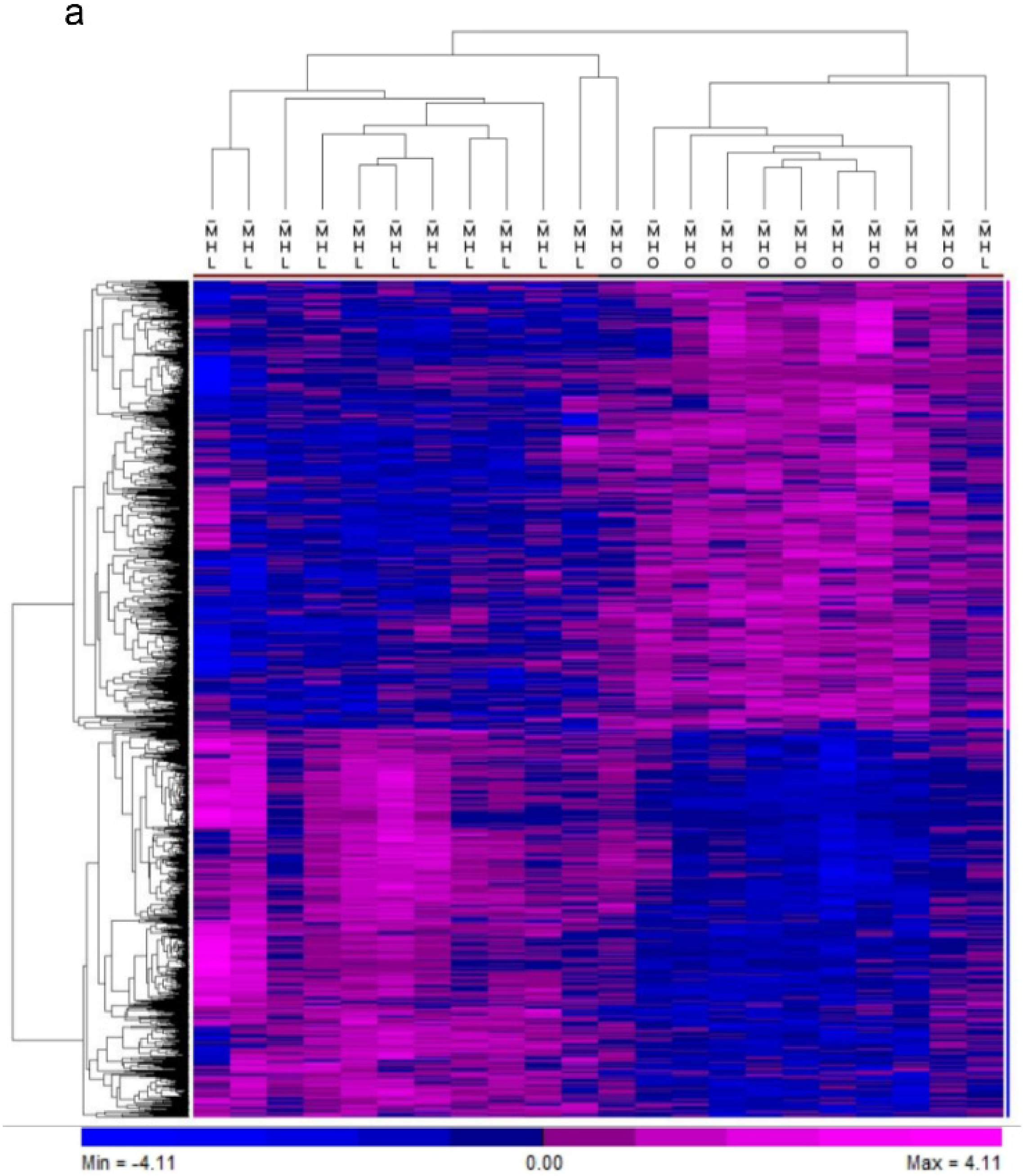

b

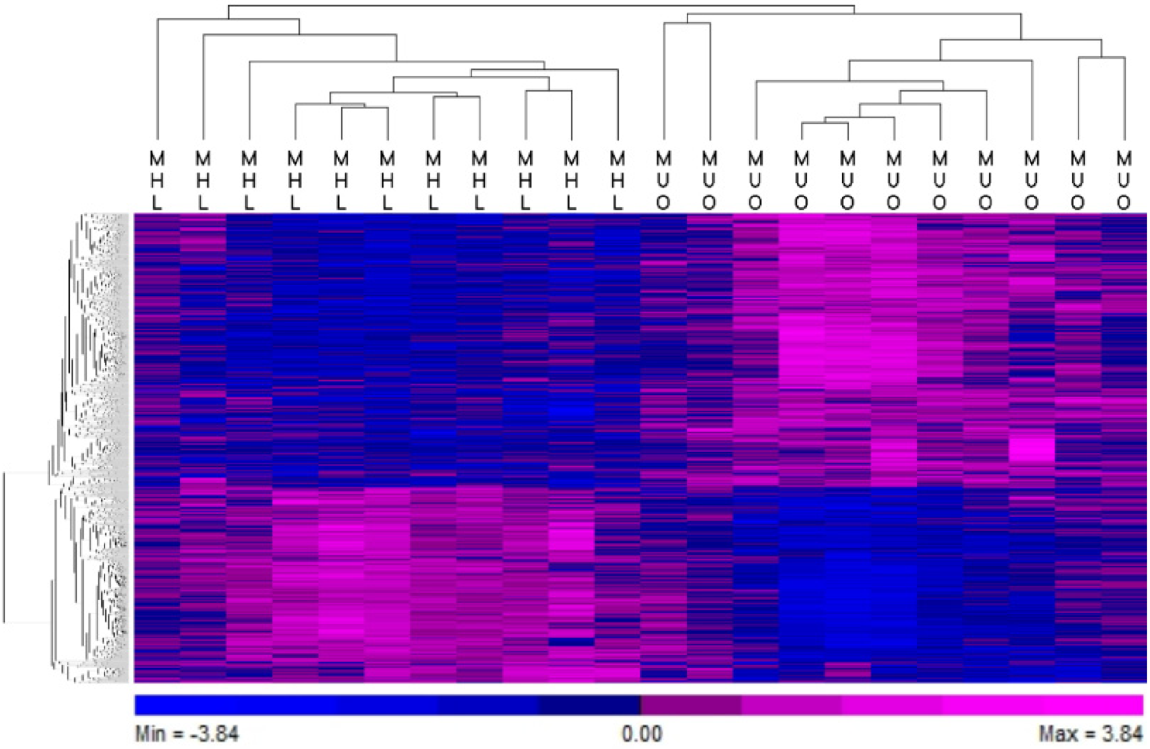

Supplementary Figure S6. Heat maps of differentially expressed genes between the two obese groups and the metabolically healthy lean (MHL) reference group. (a) A heat map of differentially expressed genes (DEGs) between the MHL (n=12) and metabolically healthy obese (MHO; n=10) groups. Purple coloration indicates gene upregulation while blue coloration indicates gene down regulation. Genes upregulated in MHO subcutaneous adipose tissue (SQ AT) are downregulated in MHL SQ AT and vice versa. Top differentially expressed gene (DEG) changes are tabulated in Table 2. Specific transcripts are not denoted in this heat map. (b) A heat map of DEGs between the MHL and the metabolically unhealthy obese (MUO; n=13) groups. The top DEGs are tabulated in Table 3. Genes upregulated in the MUO SQ AT are downregulated in the MHL SQ AT. Specific transcripts are not denoted in this heat map.

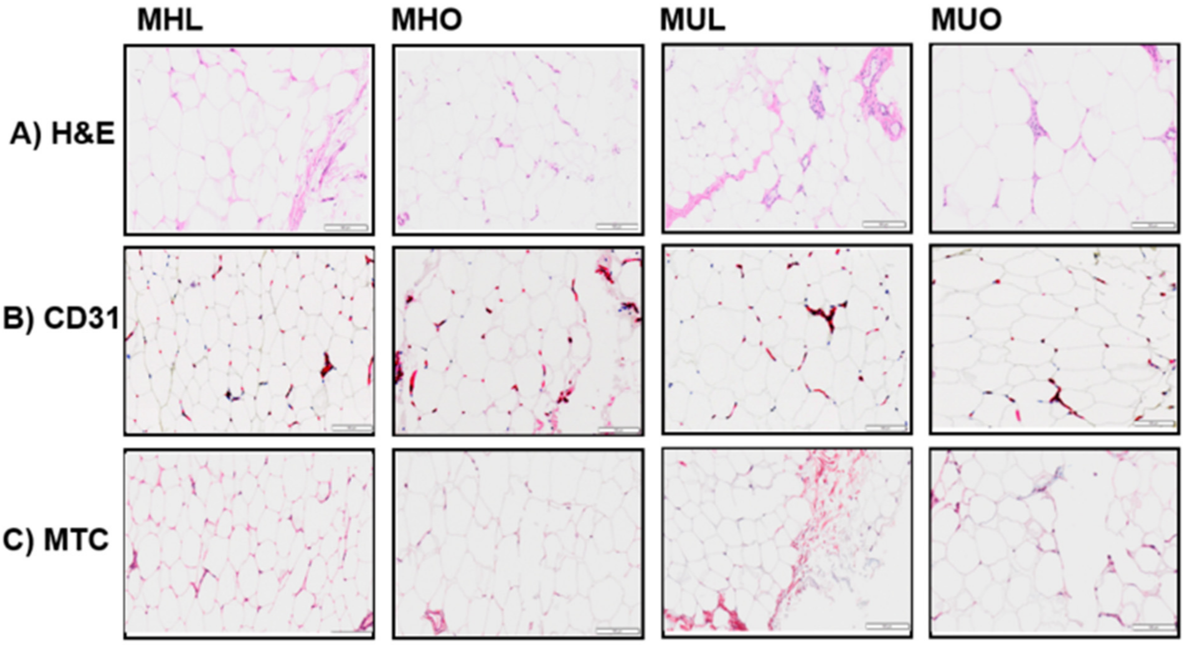

Supplementary Figure S7. No differences in adipocyte size, endothelial cell area, or fibrosis were seen between health groups. Example images from an individual from each phenotypic group of (A) hematoxylin and eosin (H&E) staining that was quantified to determine adipocyte size, (B) CD31 staining that was quantified to determine endothelial cell area, and (C) Masson's trichrome (MTC) staining that was quantified to determine tissue fibrosis. All scale bars are set to 100μm. MHL=metabolically healthy lean (n=12); MHO=metabolically healthy obese (n=10); MUL=metabolically unhealthy lean (n=9); MUO=metabolically unhealthy obese (n=13).

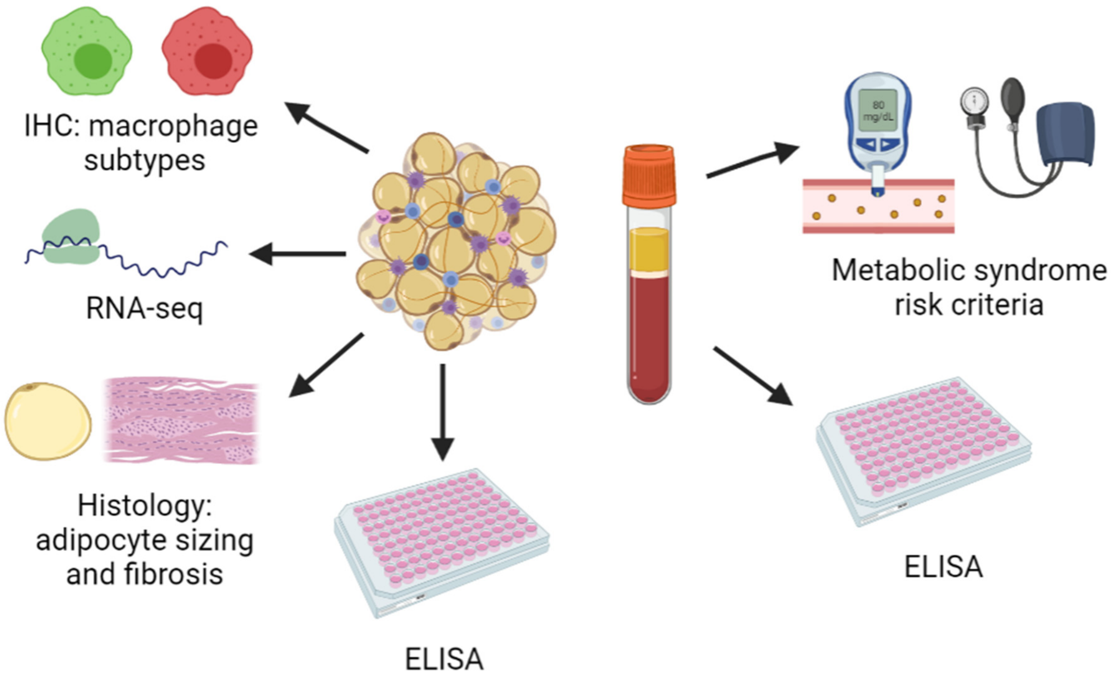

Supplementary Figure S8. Depiction of the methods performed on biological samples. Immunohistochemical analyses of macrophage subtypes, histological evaluations of adipocyte sizes and fibrosis, RNA-seq, and enzyme-linked immunosorbent assays (ELISA) were performed using collected subcutaneous adipose tissue. ELISAs and analyses of metabolic syndrome risk factors were performed using collected whole blood and isolated plasma (BioRender.com).

Measurement	Value
Waist Circumference (cm)	$\geq$ 40
Fasting Blood Glucose (mg/dL)	$\geq$ 100
Glycosylated Hemoglobin A1c (%)	$\geq$ 6
High Density Lipoprotein Cholesterol (mg/dL)	$\leq$ 50
Triglycerides (mg/dL)	$\geq$ 125
Systolic Blood Pressure (mmHg)	$\geq$ 135
Diastolic Blood Pressure (mmHg)	$\geq$ 85

Supplementary Table S1. Metabolic syndrome risk factor cut-off values for group selection. The glycaemia, dyslipidemia, and hypertension cut-off values used were equivalent to those used for humans. The waist circumference cut-off value defined obesity and was adjusted for nonhuman primates specifically. A waist circumference of >40cm corresponds with the upper 20th percentile of the vervet research colony animals [1], a percentile that corresponds with the Adult Treatment Panel III risk waist definition [1-4]. The sum of each metabolic syndrome (MetS) criterion met determined the MetS score [5]. Metabolically healthy lean animals had a MetS score of zero, metabolically healthy obese animals had a MetS score of one, and unhealthy animals had MetS scores of two or higher.

Measurement	MHL	MHO	MUL	MUO	ANOVA p-Value	Health p-Value	Obesity p-Value
Macrophage Density (cells/μm^2)	0.000008 (0.000001)	0.000009 (0.000002)	0.000006 (0.000001)	0.000007 (0.000002)	0.75	0.45	0.44
Total Cell Density (cells/μm^2)	0.000296 (0.000027)	0.000296 (0.000015)	0.000296 (0.000028)	0.000316 (0.000026)	0.91	0.68	0.69
M1 Macrophages (%)	0.68 (0.29)	0.23 (0.11)	0.29 (0.12)	1.16 (0.50)	0.18	0.35	0.65
M2 Macrophages (%)	44.27 (5.12)	62.26 (3.17)	41.14 (7.99)	51.45 (5.40)	0.06	0.23	0.03
Intermediate Macrophages (%)	2.76 (0.88)	1.75 (0.50)	0.62 (0.26)	3.03 (1.33)	0.29	0.83	0.52
Undefined Macrophages (%)	52.28 (5.77)	35.76 (3.15)	57.95 (8.19)	44.36 (5.65)	0.07	0.26	0.07

Supplementary Table S2. Subcutaneous adipose macrophage subtype distributions by group. All data are presented as means with SEM in parentheses. Macrophage density did not differ by group (metabolically healthy lean [MHL] n=12, metabolically healthy obese [MHO] n=10, metabolically unhealthy lean [MUL] n=9, metabolically unhealthy obese [MUO] n=13). Pre-determined power analyses using previously collected data indicated that we would have 80% power to detect a 1.5 standard deviation difference in M2/M1 macrophage ratio between groups with n=11 animals per group. Post-hoc power analyses using our M2/M1 macrophage ratio data and n=9 per group indicated that we achieved 100% power to detect differences in this endpoint.

Transcript	Log Fold Change	Unadjusted p-Value	Adjusted p-Value
DAPL1	-2.040	0.00724	>0.05
GJB5	-1.964	0.00059	>0.05
TSPO2	-1.957	0.00089	>0.05
ANO9	-1.607	0.00726	>0.05
CCL22	-1.514	0.00043	>0.05
SLC22A16	-1.476	0.00729	>0.05
SLC38A8	-1.381	0.00145	>0.05
EVPL	-1.304	0.00925	>0.05
CCL19	-1.298	0.00947	>0.05
PERM1	-1.271	0.00981	>0.05
SDCBP2	-1.238	0.00290	>0.05
FAM167A	-1.177	0.00520	>0.05
TPSD1	-1.145	0.00234	>0.05
ENSCSAG00000018573	-1.139	0.00793	>0.05
FGF11	-1.136	0.00029	>0.05
FRMD5	-1.021	0.00441	>0.05
CRABP1	-0.989	0.00962	>0.05
ENSCSAG00000018536	-0.966	0.00059	>0.05
ENSCSAG00000019447	-0.958	0.00727	>0.05
ANKRD9	-0.914	0.00020	>0.05
TMEM176B	-0.880	0.00871	>0.05
RAPGEFL1	-0.848	0.00894	>0.05
PRRT3	-0.706	0.00644	>0.05
EXOC3L4	-0.656	0.00561	>0.05
FN1	0.646	0.00684	>0.05
RIC3	0.663	0.00699	>0.05
PCSK4	0.671	0.00958	>0.05
NAGS	0.687	0.00767	>0.05
ASPN	0.689	0.00449	>0.05
SLC22A3	0.731	0.00241	>0.05
COL8A1	0.741	0.00712	>0.05
F2RL2	0.745	0.00184	>0.05
ENSCSAG00000003488	0.758	0.00560	>0.05
ENSCSAG00000000419	0.808	0.00287	>0.05
ENSCSAG00000018189	0.827	0.00538	>0.05
NPTXR	0.884	0.00788	>0.05
SYN2	0.909	0.00678	>0.05
ENSCSAG00000018243	0.970	0.00428	>0.05
C6orf201	0.984	0.00807	>0.05
TTPA	1.010	0.00678	>0.05
ENSCSAG00000017715	1.016	0.00317	>0.05
SLC10A6	1.133	0.00548	>0.05
ITGBL1	1.200	0.00574	>0.05
GUCY2D	1.205	0.00362	>0.05
ENSCSAG00000011474	1.245	0.00148	>0.05
IZUMO1R	1.256	0.00226	>0.05
LTBP2	1.264	0.00183	>0.05
NKX3-1	1.275	0.00263	>0.05
CA8	1.299	0.00312	>0.05
HTRA4	1.346	0.00391	>0.05
GSX2	1.357	0.00924	>0.05

P4HA3	1.365	0.00226	>0.05
SDR42E2	1.393	0.00302	>0.05
ASB5	1.454	0.00375	>0.05
ENSCSAG00000009497	1.516	0.00609	>0.05
COMP	1.724	0.00062	>0.05

Supplementary Table S3. All differentially expressed transcripts in the metabolically healthy obese (MHO) compared to the metabolically unhealthy obese (MUO) subcutaneous adipose tissue. MHO (n=10) demonstrated upregulation of nutrient transport transcripts and downregulation of inflammation-associated transcripts compared to the MUO (n=13). However, genes listed did not survive corrections for multiple corrections.

Gene ID	r-Value	p-Value
SKIDA1	0.560	0.00014
ARHGEF33	0.484	0.00134
HCN3	0.463	0.00232
NKD1	0.462	0.00234
KANSL3	0.458	0.00258
IFT172	0.441	0.00387
ZRANB3	0.440	0.00399
ZBTB37	0.415	0.00704
TTLL11	0.408	0.00810
CACNB2	0.393	0.01104
FAM8A1	0.382	0.01377
ZBTB40	0.367	0.01834
RWDD4	0.354	0.02311
TBC1D8B	0.348	0.02590
TRDMT1	0.337	0.03123
ABCC2	0.315	0.04483
ARPC5L	-0.303	0.05393
REEP4	-0.324	0.03867
CNKSR1	-0.374	0.01592
HSPA14	-0.385	0.01282
KIFC3	-0.389	0.01203
IGHD	-0.398	0.00988
GRID2IP	-0.409	0.00796
SEPTIN4	-0.416	0.00682
CDKN1A	-0.419	0.00646
LRRC3C	-0.420	0.00621
ZDHHC5	-0.421	0.00612
FOSL1	-0.427	0.00540
FAM160A2	-0.428	0.00526
KCNG1	-0.429	0.00515
MS4A1	-0.430	0.00500
RDH16	-0.435	0.00451
HSF4	-0.436	0.00436
ALAS1	-0.469	0.00200
RAB20	-0.555	0.00017

Supplemental Table S4. Statistically significant associations between gene-specific transcript expression levels and the subcutaneous adipose tissue M2/M1 ratio (n=43). Associations were considered when transcripts were detectable in $\geq$ 21 of the animal subjects. Pearson correlations were used to determine associations.

Measurement	Sample	MHL	MHO	MUL	MUO	ANOVA p-Value	Health p-Value	Obesity p-Value
MCP-1 (pg/mL)	Plasma	353.09 (70.73)	349.60 (34.11)	315.42 (33.04)	277.13 (29.11)	0.61	0.35	0.92
Adiponectin (ng/mL)	Plasma	66.50 (8.99)	73.90 (9.45)	99.32 (11.16)	71.76 (14.05)	0.17	0.07	0.59
PAI-1 (ng/mL)	Plasma	6.66 (1.96)	7.01 (1.79)	2.95 (0.57)	8.84 (2.47)	0.36	0.80	0.21
IL-6 (pg/mL)	Plasma	5.28 (0.74)	10.39 (3.18)	7.44 (2.80)	7.20 (1.74)	0.35	0.93	0.93
IL-1β (pg/mL)	Plasma	1.61 (0.37)	3.28 (0.79)	3.74 (1.04)	3.43 (1.14)	0.48	0.55	0.91
MCP-1 (pg/mL)	SQ AT	3.98 (0.80)	8.52 (2.75)	6.72 (2.11)	10.98 (2.76)	0.24	0.06	0.02*
IL-10 (pg/mL)	SQ AT	53.47 (8.28)	24.23 (4.64)	46.12 (8.59)	27.05 (4.05)	0.03*	0.99	0.004*
TGFβ (pg/mL)	SQ AT	1766.38 (93.82)	1844.47 (73.54)	2220.74 (194.39)	1847.16 (66.42)	0.09	0.08	0.38

Supplementary Table S5. Cytokines measured in circulation and in protein extracted from subcutaneous adipose explants. All data are presented as means with SEM in parentheses (metabolically healthy lean [MHL] n=12; metabolically healthy obese [MHO] n=10; metabolically unhealthy lean [MUL] n=9; metabolically unhealthy obese [MUO] n=13). Circulating cytokines did not differentiate groups. However, tissue measures of interleukin-10 (IL-10) differed between groups (overall ANOVA p=0.03) and by obesity status (p=0.004). Tissue levels of monocyte chemoattractant protein-1 (MCP-1) also differed by obesity status (p=0.02), while transforming growth factor β (TGF-β) demonstrate a trend toward differing between groups (overall ANOVA p=0.09) and by health status (p=0.08).

Measurement	MHL	MHO	MUL	MUO	ANOVA p-Value	Health p-Value	Obesity p-Value
% Positive CD31	1.08 (0.15)	1.21 (0.23)	1.27 (0.18)	1.41 (0.20)	0.64	0.54	0.59
% Positive MTC	0.08 (0.015)	0.10 (0.02)	0.76 (0.64)	0.10 (0.019)	0.39	0.37	0.50

Supplemental Table S6. Tissue vasculature and fibrosis did not differ by group, health or obesity status. All data are presented as tissue positive staining area means with SEM in parentheses (metabolically healthy lean [MHL] n=12; metabolically healthy obese [MHO] n=10; metabolically unhealthy lean [MUL] n=9; metabolically unhealthy obese [MUO] n=13).

Measurement	MHL	MHO	MUL	MUO	p-Value
Relatedness Coefficient	0.0118	0.0171	0.00977	0.0156	>0.05

Supplemental Table S7. The familial relational coefficients of animals in each metabolic health group. All data are presented as medians (metabolically healthy lean [MHL] n=12; metabolically healthy obese [MHO] n=10; metabolically unhealthy lean [MUL] n=9; metabolically unhealthy obese [MUO] n=13). The animals' relatedness in each group was not significantly different.

References

1. Kavanagh, K., et al., Characterization and heritability of obesity and associated risk factors in vervet monkeys. 2007. 15(7): p. 1666-1674.
2. Detection, N.C.E.P.E.P.o. and T.o.H.B.C.i. Adults, Third report of the National Cholesterol Education Program (NCEP) Expert Panel on detection, evaluation, and treatment of high blood cholesterol in adults (Adult Treatment Panel III). 2002: The Program.
3. Lean, M., T. Han, and C.J.B. Morrison, Waist circumference as a measure for indicating need for weight management. 1995. 311(6998): p. 158-161.
4. Palaniappan, L., et al., Predictors of the incident metabolic syndrome in adults: the Insulin Resistance Atherosclerosis Study. 2004. 27(3): p. 788-793.
5. Kavanagh, K., et al., Regulators of mitochondrial quality control differ in subcutaneous fat of metabolically healthy and unhealthy obese monkeys. Obesity, 2017. 25(4): p. 689-696.
